# Issues in Using Human Variability Distributions to Estimate Low-Dose Risk

**DOI:** 10.1289/ehp.0901250

**Published:** 2009-10-23

**Authors:** Kenny S. Crump, Weihsueh A. Chiu, Ravi P. Subramaniam

**Affiliations:** 1 Louisiana Tech University, Ruston, Louisiana, USA; 2 National Center for Environmental Assessment, Office of Research and Development, U.S. Environmental Protection Agency, Washington, DC, USA

**Keywords:** dose-response model, human variability distribution, log-normal distribution, low-dose risk, risk assessment, threshold distribution

## Abstract

**Background:**

The National Research Council (NRC) Committee on Improving Risk Analysis Approaches Used by the U.S. EPA (Environmental Protection Agency) recommended that low-dose risks be estimated in some situations using human variability distributions (HVDs). HVD modeling estimates log-normal distributions from data on pharmacokinetic and pharmacodynamic variables that affect individual sensitivities to the toxic response. These distributions are combined into an overall log-normal distribution for the threshold dose (dose below which there is no contribution to a toxic response) by assuming the variables act independently and multiplicatively. This distribution is centered at a point-of-departure dose that is usually estimated from animal data. The resulting log-normal distribution is used to quantify low-dose risk.

**Objective:**

We examined the implications of various assumptions in HVD modeling for estimating low-dose risk.

**Methods:**

The assumptions and data used in HVD modeling were subjected to rigorous scrutiny.

**Results:**

We found that the assumption that the variables affecting human sensitivity vary log normally is not scientifically defensible. Other distributions that are equally consistent with the data provide very different estimates of low-dose risk. HVD modeling can also involve an assumption that a threshold dose defined by dichotomizing a continuous apical response has a log-normal distribution. This assumption is shown to be incompatible (except under highly specialized conditions) with assuming that the continuous apical response itself is log normal. However, the two assumptions can lead to very different estimates of low-dose risk. The assumption in HVD modeling that the threshold dose can be expressed as a function of a product of independent variables lacks phenomenological support. We provide an example that shows that this assumption is generally invalid.

**Conclusion:**

In view of these problems, we recommend caution in the use of HVD modeling as a general approach to estimating low-dose risks from human exposures to toxic chemicals.

Current guidelines of the U.S. Environmental Protection Agency (EPA) for risk assessment differentiate carcinogenic and noncarcinogenic responses. Quantitative estimates of risk are not developed for noncarcinogenic responses; instead, reference doses (RfDs), estimates of daily exposures to the human population that are assumed to be without appreciable risk of adverse effects, are calculated by dividing a point-of-departure (POD) dose by factors to account for extrapolation from experimental animals to humans, variability within the human population, and data limitations (e.g., U.S. EPA 1991). With cancer, different approaches are applied depending on the perceived mode of action (MOA). If there is sufficient evidence that the MOA leading to cancer is nonlinear, then the assessment does not provide a quantitative estimate of risk but proceeds in a manner similar to that for noncarcinogens. Otherwise, linear extrapolation from the POD to low doses is used to quantify low-dose risk (U.S. EPA 2005). This bifurcated approach has been criticized as being unjustified because there is no clear evidence that the dose responses for cancer and other toxicities are fundamentally different (e.g., Bogdanffy et al. 2001). Also, there is concern that having quantitative estimates only for cancer has led to an overemphasis on this response at the expense of other serious hazards (e.g., Crump et al. 1997).

Another perceived shortcoming of current methods is that quantitative results, whether RfDs for noncarcinogens or risk estimates for carcinogens, are presented as point estimates (i.e., single values), even though it is generally recognized that considerable uncertainty exists in these estimates. Although a qualitative discussion of the uncertainty is presented, it is difficult to translate this discussion into quantitative terms. Several proposals have been made to quantify uncertainty in risk assessments through the use of probability distributions [see, e.g., National Research Council (NRC) 2008 and references therein]. Such an approach could give decision makers a much better basis for decision making than would be obtained from having only a single-point estimate.

The NRC Committee on Improving Risk Analysis Approaches Used by the U.S. EPA addressed these and other issues by developing far-reaching recommendations for changing how the U.S. EPA assesses risk. The committee report, titled *Science and Decisions,* (NRC 2008), contains recommendations aimed at improving both the utility of risk assessments for decision making and the technical analysis in risk assessment. These latter recommendations include ones for probabilistic characterization of risk that involve explicit characterization of human heterogeneity and harmonizing the treatment of carcinogens and noncarcinogens.

The recommendation in *Science & Decisions* for harmonizing cancer and noncancer risk assessment involves a paradigm for quantitative risk assessment composed of three conceptual models that envision three categories of dose responses derived from mechanistic considerations: conceptual model 1, threshold dose response at the individual level and linear response at the population level; conceptual model 2, threshold response at the individual level and nonlinear response at the population level; and conceptual model 3, linear response at both the individual and the population level. These models are proposed for use by the U.S. EPA in extrapolating dose–response relationships to increases in risks that are lower than those that can be measured directly in most studies (e.g., 10^−5^). Each of these models provides a probabilistic description of the dose response. However, determining which conceptual model is appropriate in a particular situation would involve understanding the “underlying biologic processes and how they contribute to an individual’s dose–response relationship, the nature of human variability, and the degree to which the processes may be independent of background exposures and processes” (NRC 2008).

This paradigm represents a significant departure from current U.S. EPA practice. Among the changes incorporated in this recommendation are *a*) providing quantitative low-dose–extrapolated risk estimates not only for cancer, as is currently done, but for all types of health effects; *b*) basing the quantitative approach not on the type of toxic effect (whether cancer or not), but on consideration of the perceived individual dose responses, the nature of human variability, and how the toxic substance interacts with background processes that contribute to background toxicity; *c*) not restricting linear extrapolation to carcinogenic responses but applying it to some noncarcinogenic responses as well; and *d*) providing not just a single estimate of risk but a probabilistic description.

We offer our congratulations to the *Science & Decisions* (NRC 2008) committee on their very insightful report, which we believe contains many useful recommendations, particularly the recommendation to harmonize risk assessment for cancer and noncancer effects. Modifying guidelines to implement the committee’s recommendations and implementing the resulting guidelines would be a major undertaking. More important, such changes would constitute a major paradigm shift for the U.S. EPA. Before proceeding on this course, which has far-reaching implications, we believe it is prudent to look carefully at the proposals to determine if they are scientifically credible and operationally feasible. We offer this article as a contribution to that process.

In this study we focused on the part of the proposed risk assessment paradigm identified as conceptual model 2. The dose responses for toxic effects that fall under this model are quantified using human variability distributions (HVDs). Our evaluation of this approach has identified some serious conceptual and operational problems.

## Low-Dose Extrapolation Using HVDs

Conceptual model 2 in *Science & Decisions* (NRC 2008) employs HVDs to estimate the dose response from exposure to a toxin (HVD modeling) and uses this dose response to extrapolate risk to low doses. The approach includes the following three features:

- As explained in *Science & Decisions* (NRC 2008, box 5–3), a median human POD, accompanied by a lower bound for the POD, is determined from chemical-specific animal bioassay data and (usually) nonchemical-specific data on differences in animal and human sensitivity. The POD is interpreted as the human dose that would cause 50% of humans to respond adversely among those who would otherwise not have the response.
- A log-normal distribution representing human interindividual variability is used to extrapolate from the POD down to low risk levels (e.g., 10^−5^). The median of this log-normal distribution is set equal to the POD, which determines the log mean. The log variance is determined from fitting log-normal distributions to different sets of data representing human variability in various pharmacokinetic and pharmacodynamic variables that are considered to be associated with individual variability in response to the toxin.
- The pharmacokinetic and pharmacodynamic variables are assumed to interact mutually independently and multiplicatively, so that the overall distribution of human interindividual variability is log normal with log variance equal to the sum of the log variances of the individual sources of human variability.

The resulting mathematical expression for the risk from a dose, *D*, can be expressed mathematically as


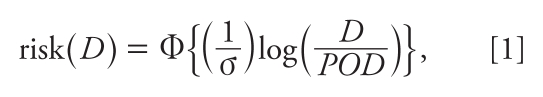


where Φ is the standard normal distribution function, log indicates base 10 logarithm, and σ^2^ is the overall log variance (the sum of the log variances of the individual sources of variation). Our focus here is on the third step in this process: the use of the distribution of human interindividual variability to represent the dose response.

*Science & Decisions* (NRC 2008) does not provide details regarding the individual steps in HVD modeling. However, the document refers extensively to the model developed by Hattis and colleagues (Hattis et al. 1999, 2002; Hattis and Goble 2007), and the illustrative example provided in *Science & Decisions* relies on this model. The Hattis model is a detailed model that has been developed over many years, and a large amount of data has been collected and analyzed in its support. Consequently, we consider the Hattis model to represent HVD modeling, although our conclusions apply more widely.

We need to specify what “risk” is represented by Expression 1. Because this expression approaches zero as *D* approaches zero and because many adverse effects can occur in unexposed individuals, it does not apply to *P*(*D*), the probability of responding when exposed to dose *D*. Also, although the “additional risk” [the probability of disease with dose *D* minus the corresponding probability when unexposed, *P*(*D*) – *P*(0)] is zero when *D* = 0, when background risk is present [*P*(0) > 0], it is not a true probability distribution because, unlike Expression 1, the additional risk does not approach 1 as *D* becomes large. Consequently, we believe the best interpretation of Expression 1 is that it represents “extra risk”—the additional risk divided by the probability of remaining disease free when unexposed {[*P*(*D*) – *P*(0)] ÷ [1 – *P*(0)]}.

### Details of the HVD model

The HVD model, as formulated in Hattis et al. (1999), specifically considers the following pharmacokinetic and pharmacodynamic variables (called parameters by Hattis and colleagues) that can induce variability in overall human response: *a*) contact rate (e.g., breathing rate ÷ body weight, fish consumption ÷ body weight), *b*) uptake or absorption (milligrams per kilogram ÷ intake or contact rate, *c*) general systemic availability net of first-pass elimination and dilution via distribution volume, *d*) dilution via distribution volume, *e*) systemic elimination ÷ clearance or half-life, *f*) active-site availability ÷ general systemic availability, *g*) physiologic variable change ÷ active site availability, and *h*) functional reserve capacity (i.e., change in baseline physiologic variable needed to pass a criterion of abnormal function).

Some of these variables are further subclassified by exposure route and age. In most cases the available data do not inform variability from a single variable but from a combination of variables involved in the steps from exposure to apical response. For example, measurements of serum levels of a toxin resulting from measured air concentrations would provide information on the variability induced by variables *a*–*e* in the above list, and data on the apical response as a function of serum measurements would provide information on variability induced by variables *f*–*h*. Estimates of the individual log variances associated with each of the eight variables are developed from a regression model based on the assumptions of log normality and that the total variance can be obtained by adding the variances associated with each individual variable (Hattis et al. 1999).

The pharmacodynamic variables (*g* and *h* in the above list) are quantified using a tolerance distribution (i.e., by specifying the probability that an individual is affected as a function of dose). Such a distribution can be thought of as a distribution of individual threshold doses; if an individual is affected when exposed to a given dose, that means his or her threshold was less than the given dose, and otherwise the threshold exceeded the given dose. Thus, the probability that an individual is affected is the same as the probability that his or her threshold was less than the given dose. In HVD modeling, the tolerance distribution (i.e., distribution of individual thresholds) is assumed to have the log-normal form of Expression 1, which, if the POD is identified with a risk of 0.5, can be written as Φ {[log(*D*) – μ]/σ}, where μ and σ^2^ are the log mean and log variance of the threshold dose. This distribution is often fitted to binary response data in the equivalent log-probit form, Φ[*a* + *b*log(*D*)], where *b* = 1/σ and *a* = –μ/σ. In HVD modeling, σ^2^ represents the combined variance of all of the steps assumed to lead from exposure to response, and the contributions to this variance by individual steps are estimated by the regression approach described above.

### Uncertainty in low-dose extrapolation using HVD modeling

Thirty years ago numerous papers pointed out that different dose–response models could describe toxicity data about equally well in the high-dose range, but they could differ by orders of magnitude at low doses (see NRC 1983). The identical problem occurs with HVD modeling. There is no convincing basis for assuming that human distributions of adverse responses are log normal. (We address claims of support for the log-normal distribution in the next section.) In this section, we show that other distributions will fit the data about the same but provide estimates of low-dose risk that differ by many orders of magnitude.

**Figure 1:**
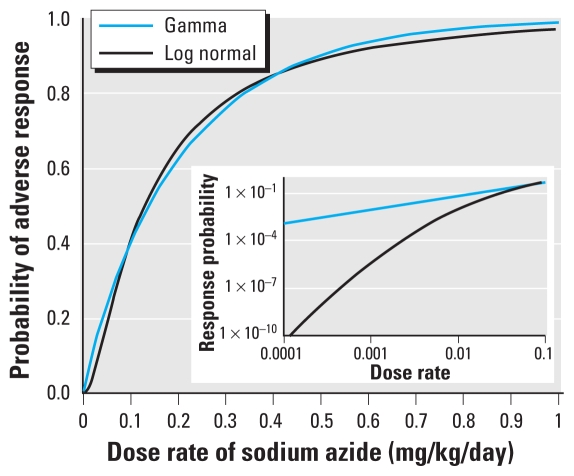
Comparison of a gamma distribution with the log-normal distribution recommended by the NRC committee (NRC 2008, box 5–3) for estimating low-dose risks of sodium azide. Inset shows the same plot on a log-log scale showing low-dose divergence.

Figure 1 compares the log-normal distribution in the *Science & Decisions* example used to illustrate HVD modeling (NRC 2008, box 5–3) with the gamma distribution with probability density function (Johnson et al. 1991)


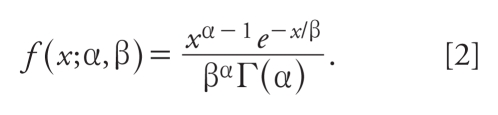


The gamma and log-normal distributions agree fairly closely through most of the observable range but diverge significantly in the tails. Based on the log-normal curve shown in Figure 1, *Science & Decisions* reports a median human dose corresponding to a risk of 10^−5^. At this same dose, the risk estimated using the gamma distribution shown in Figure 1 is 10^−2^, a thousand-fold higher.

The gamma distribution was selected for this comparison simply for mathematical convenience. There is no convincing reason that an HVD should follow any particular distribution (see next section). Considering the universe of possible distributions, it is clear that there are distributions that match the log normal better than the gamma in the observable range (e.g., for risks ≥ 0.01), including distributions that replicate the log normal exactly in this range but still diverge from the log normal at lower risks. (e.g., consider a distribution that matches the log normal for risks ≥ 0.01 but decreases continuously from 0.01 to zero at a dose equal to 0.5 of the dose corresponding to a risk of 0.01.)

Data sets used to estimate the log-normal variances for HVD modeling often contain a fairly limited number of observations, which suggests that a wide variety of probability distributions will often describe these data adequately. To illustrate this point, we fit the log-normal distribution, gamma distribution (Expression 2), and shifted log-gamma distribution (the distribution of *e^X^* – 1 where *X* is gamma distributed) to 198 sets of data collected and used by Hattis et al. (2002) to model human variability in pharmacokinetic variables (Table 1). The gamma distribution provided the best fit [i.e., smallest Akaike information criterion (AIC); Akaike 1974] to 77 of these data sets (39%), the shifted log-gamma distribution provided the best fit to 83 data sets (42%), and the log-normal distribution provided the best fit only to 38 data sets (19%). In most instances the differences among the AIC criteria were very small. Nevertheless, this analysis illustrates the notion that, as one would expect, there is nothing unique about the ability of the log-normal distribution to fit HVD data, and distributions other than the log normal will typically fit such data as well or better than the log-normal distribution.

## Use of the Log-Normal Distribution in HVD Modeling

*Science & Decisions* contains no justification for the recommendation that a log-normal distribution be used in HVD modeling. In an informal perusal of the literature, we uncovered two lines of argument in support of the log normal. By far the most often cited was that a log-normal distribution often fits data on human variability (e.g., Hattis and Goble 2007). However, nowhere did we find an examination of the fits of other distributions, and, as illustrated above, other distributions can fit as well or better than the log normal. Consequently, providing an adequate fit to data is not a sufficient justification for exclusive use of the log-normal distribution.

A second argument sometimes mentioned in support of the log-normal distribution is based on the central limit theorem, which implies that under certain regularity conditions the distribution of a product of random variables will approach log normality as the number of terms increases. For example, Hattis et al. (1999) stated, “Such a [log-normal] distribution would be expected if there are many factors, each contributing modestly to the individual variability in threshold doses, and if each factor tends to act multiplicatively to affect individual thresholds.” However, no phenomenological model has been provided to explain how the quantitative effect of multiple factors on risk could be expressed as a product of the individual factors, nor have any such potential factors even been identified. Without any such evidence that the central limit theorem is applicable, the theorem does not provide any theoretical support for the log-normal distribution. Moreover, in a following section we present a simple yet plausible phenomenological model in which two factors interact to produce an adverse effect but do so neither multiplicatively nor independently, both of which are conditions needed for application of the central limit theorem. Thus, we find reliance on the central limit theorem to support the exclusive use of the log-normal distribution in HVD modeling to be very tenuous, especially given its use for low-dose extrapolation, as discussed next.

Note that the central limit theorem is a limit theorem. Even if the necessary conditions for applying the theorem were met (which, as noted above, seems unlikely), the resulting HVD distribution would be only approximately log normal. The divergence from the log-normal distribution would be greatest (on a percentage basis) in the tails of the distribution, which is the range of interest in low-dose extrapolation. Thus, even if the central limit theorem were applicable, and even if enough physiologic variables were multiplied together to obtain a reasonably good approximation to the log-normal distribution in the observable range, the tails of the distribution could still be very poorly described by the log-normal distribution.

**Figure 2:**
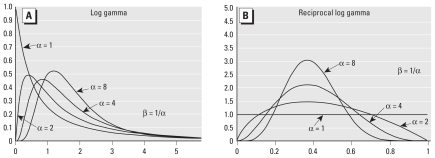
Graphs of log-gamma (*A*) and reciprocal log-gamma (*B*) probability densities: probability densities of *e^X^* and *e*^−^*^X^*, respectively, where *X* has a gamma distribution with shape parameter α and scale parameter β.

To investigate the accuracy of the log-normal approximation (i.e., the log-normal distribution whose log transform has the same mean and variance as the log transform of the true distribution) in predicting the lower tail of the HVD distribution, we consider two theoretical true distributions: “log gamma” and “reciprocal log gamma” (Figure 2), which are the distributions of *e^X^* and *e*^−^*^X^*, respectively, where *X* has a gamma distribution. The sum of independent random variables having gamma distributions with a common α value also has a gamma distribution with the same α value and a β value equal to the sum of the individual β values. This feature of the gamma distribution facilitates numerical calculations by making it possible to compute the distribution of sums of independent gamma distributions with a common α value and, correspondingly, the product of independent log gamma distributions or reciprocal log-gamma distributions having a common α value, using only the incomplete gamma function and elementary functions.

**Figure 3:**
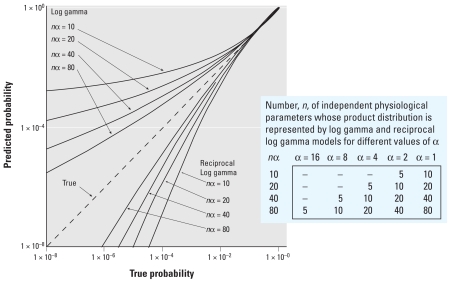
Comparison of cumulative probability distribution of the product of *n* independent variables with log-gamma or reciprocal log-gamma distributions (true probability) with the log-normal approximation to the true probability distribution. The table shows the number *n* of independent physiologic parameters whose product distribution is represented by log-gamma and reciprocal log-gamma models for different values of α.

Figure 3 compares log-gamma and reciprocal log-gamma distributions with the corresponding log-normal approximations for different values of the shape parameter, α, in the gamma distribution. The true probability is plotted on the horizontal axis, and the corresponding log-normal approximation on the vertical axis. (These curves are independent of β.) As indicated by the table in Figure 3, each curve has multiple interpretations. For example, the curve corresponding to “reciprocal log gamma *n*α = 20” shows the error in the log-normal approximation to the distribution of *a*) the product of five reciprocal log-gamma variables, each with shape parameter α = 4 (and a common β); *b*) the product of 10 reciprocal log-gamma variables having a shape parameter α = 2; or *c*) the product of 20 reciprocal log-gamma variables having a shape parameter α = 1.

In each case depicted in Figure 3, the log-normal approximation matches the exact curve closely for probabilities between 0.1 and 0.9 but deviates considerably from the true value in the lower tail. The errors in the approximation can be in the direction of either overestimating (illustrated by the log-gamma model) or underestimating (illustrated by the reciprocal log-gamma model) low-dose risk. For example, with the reciprocal log-gamma model, α = 2, and 10 terms in the product (*n*α = 20), when the true risk is 10^−5^ the log-normal approximation predicts a risk of 10^−8^.

Note that the results shown in Figure 3 depict absolutely best cases: *a*) The HVD is assumed to result from the product of a large number (*n*) of individual physiologic variables that contribute to human variation (5 ≤ *n* ≤ 80), *b*) all of these variables are assumed to have the same distribution, and *c*) these variables are assumed to be independent. Any deviation from these assumptions would be expected to degrade the log-normal approximation. None of these assumptions appears to be even approximately valid, including, in particular, the basic assumption that human variability can be reasonably described by a product of individual variables acting independently (addressed in a following section). But, as Figure 3 demonstrates, even in these theoretical best cases, the log-normal approximation can be in error by as much as several orders of magnitude in the low-risk range of interest (10^−6^ to 10^−3^).

### Threshold dose and apical response cannot both be log normal

HVD modeling assumes that the threshold dose is log normally distributed. Alternatively, it could be assumed that the apical response on which the threshold dose is based is log normally distributed. In fact, it seems to us that, if the central limit theorem argument for log normality has any credence, it would more likely apply to the apical response than to the threshold dose defined from the apical response. In this section, we show that these two assumptions are incompatible: if the underlying continuous apical response has a log-normal distribution, then the threshold (tolerance) distribution based on the apical response cannot be log normal, except under highly specific circumstances. We also examine the differences in low-dose risk implied by these two assumptions.

Suppose an apical response, *X*, has a log normal distribution with median *m*(*D*) and log variance σ^2^. This distribution may be written as


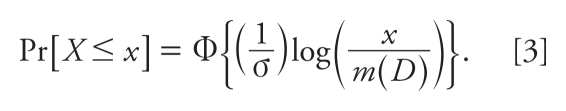


Further, suppose that the apical response is dichotomized using a cut point *A*, and assume for definiteness that larger values of *X* are more adverse. For example, if the threshold dose is defined in terms of some continuous response as the dose that results in a response equal to 50% of the maximal possible response (Hattis et al. 1999), then in this case the apical response at a dose is the percentage of maximal response and the cut point *A* is 50%. The corresponding tolerance distribution (distribution for the threshold dose *D_T_* defined by the cut point *A*) is as follows (see also Appendix):


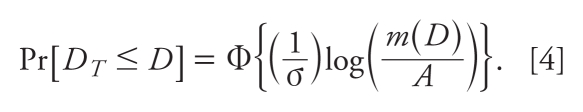


This distribution, although it appears similar to Expression 3, is a function of dose *D*, whereas Expression 3 is a function of *x*, the value of the apical response. Thus, this distribution is generally not log normal [an exception would be the special case in which σ does not depend upon *D* and *m*(*D*) is proportional to *D*]. A similar observation was made by West and Kodell (1999) with respect to the normal distribution. Consequently, as we started out to show, in general the apical response and the threshold dose defined by dichotomizing the apical response cannot both be log normally distributed.

The expression for extra risk corresponding to Expression 4 is


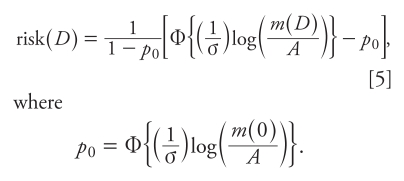


where


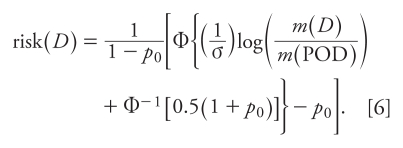


By identifying the POD with a 50% extra risk [risk(POD) = 0.5], the cut point *A* can be solved in terms of POD and other quantities. The result is as follows (see also Appendix):


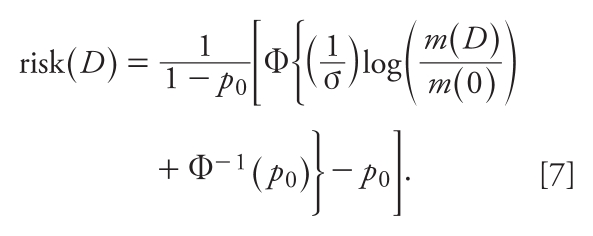


If, as is often the case, the apical response can be positive in the absence of dose [*p*_0_ > 0 or, equivalently, *m*(0) > 0], Expression 6 can be rewritten in the equivalent form (see also Appendix):

There are two important differences between Expressions 6 and 7 for risk, which assume the apical response has a log-normal distribution, and Expression 1 for risk, which is used in HVD modeling. First, in Expressions 6 and 7 the risk depends upon the median *m*(*D*) of the apical response, whereas in Expression 1 it does not. Second, from Expression 7 it is clear that the low-dose risk depends on the behavior of Φ in the neighborhood of Φ^−1^(*p*_0_), whereas in Expression 1 the low-dose risk depends on the behavior of Φ in the neighborhood of zero. Thus, these two expressions can have very different consequences for low-dose risk. Table 2 illustrates these consequences by comparing estimates of risk calculated using HVD modeling (Expression 1) with estimates based on Expression 7.

### HVD variables do not act independently and multiplicatively

As noted above, in HVD modeling the log variances of the individual (assumed log normally distributed) variables that contribute to variability are summed to obtain the overall log variance. This assumes that different variables are independent and that the overall effect of these variables can be represented by the product of the individual variables (or reciprocals of these variables, since the log variance of a variable is the same as that of its reciprocal) or, equivalently, by the sum of their logarithms. We are not aware of any mechanistic support for these assumptions. As demonstrated in the following simple example, they do not appear to be valid in general.

Suppose that data are available on an apical response *X* as a function of blood serum concentration *C* of a toxin rather than the external dose. If this response is dichotomized to form a tolerance distribution for the concentration threshold, as is often done in HVD modeling, the probability of an adverse response as a function of *C* would thus correspond to Expression 1 using serum concentration rather than external dose, as follows:


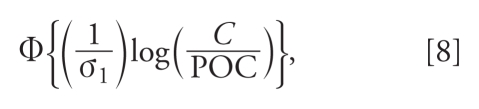


where σ_1_^2^ is the total variance associated with the steps between blood concentration and apical response, and POC is the blood concentration corresponding to 50% response. Further, assume a constant external dose rate *D* and first-order elimination of the toxin. Under these conditions, the serum concentration *C* is proportional to the product of the external dose *D* and the biological half-life *T*, that is, *C* = *KTD*, where *K* is constant. We make the assumption of HVD modeling that *T* is distributed log normally in the population, with log mean μ*_T_* and log variance σ_2_^2^. Based on Expression 8 and the assumptions regarding *C* and *T*, the probability of an adverse response is given by (see also Appendix)


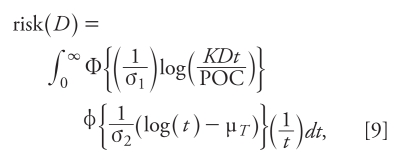


where φ is the standard normal probability density function. This expression is different from what would be predicted by HVD modeling (Expression 1 with σ^2^ = σ_1_^2^ + σ_2_^2^). Moreover, in this example the threshold serum concentration is not independent of the half-life *T*, as assumed in HVD modeling.

If, rather than assuming the threshold serum concentration to have a log-normal distribution, the continuous apical response upon which it is based is assumed to be log normal with median *m*(*C*) and variance σ^2^, the expression for risk is equal to (see Expression 6 and Appendix)


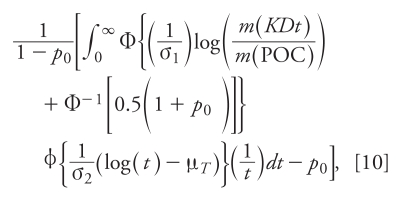


which also is different from Expression 1. In this case as well, the expression for risk (Expression 10) is not a log-normal distribution, as assumed by HVD modeling, and *X* and *T* are not independent.

This demonstrates that, in general, critical assumptions implicit in HVD modeling are not valid. These assumptions are that the various physiologic variables included in the model are independent and that the joint effect of multiple variables that affect risk can be represented by the product of the individual variables. Moreover, we know of no realistic and nontrivial phenomenological model that does satisfy these conditions.

## Discussion

The NRC (2008) found “substantial deficiencies” in the current approaches to the treatment of uncertainty and variability in quantitative risk assessment of both cancer and noncancer outcomes. To address these concerns, they recommended three conceptual models for estimating low-dose risk. In all of these models, clear separation is maintained between *a*) estimation in the range of observation (e.g., the derivation of a human POD using benchmark dose modeling and probabilistic cross-species, route, or duration adjustments) and *b*) extrapolation to low doses. The focus of our analysis is on the reliability of low-dose extrapolation using models derived from HVD modeling, recommended by “conceptual model 2” in *Science & Decisions* (NRC 2008). We conclude that the prospects for improving (i.e., reducing uncertainty in) estimates of low-dose risk using HVD modeling are not encouraging, for several reasons.

First, low-dose extrapolation of risks predicted by HVD modeling is unreliable due to model uncertainty. Although the log-normal model is mathematically convenient, there is no scientific justification for assuming that HVDs are log normal. Moreover, other distributions will describe the available data equally well but provide low-dose risk estimates that differ by many orders of magnitude from those obtained under the log-normal assumption. Therefore, although estimating human variability in the range of the observed data may not be sensitive to the choice of distributions, so use of the log-normal distribution for that purpose may be adequate, there is likely to be high uncertainty in any significant extrapolation to the tail of the distribution.

Second, in HVD modeling the log-normal expression for risk as a function of applied dose (Expression 1) is best interpreted as the conditional distribution of threshold doses given that the threshold is positive (i.e., as extra risk). These thresholds are often derived by dichotomizing a continuous or graded apical response. Although the log-normal assumption would seem to apply more naturally to the apical response itself, a log-normal distribution for the apical response is incompatible with a log-normal distribution for the threshold (except under highly restrictive conditions). However, assuming that the continuous apical responses are log normal can lead to very different risk estimates than assuming the derived thresholds are log normal (Table 2).

Third, the assumption in HVD modeling that risk can be expressed as a function of a product of independent variables lacks phenomenologic support. Here we present a biologically plausible model in which the two variables affecting risk interacted neither multiplicatively nor independently. Consequently, this assumption appears to be generally invalid. This assumption may not have much impact in the range of observation if it leads to an adequate description of the data, but its lack of support calls into further question the reliability of its use for low-dose extrapolation.

In view of these problems, we recommend caution in the use of HVD modeling to set exposure standards for human exposures to toxic chemicals. Special cases in which application of HVD modeling to estimate low-dose risk may be reasonable, if such cases exist, remain to be identified.

Whereas this paper focuses on conceptual model 2 that employs HVD modeling, *Science & Decisions* also proposes the use of conceptual models 1 and 3 under certain conditions. Both of these models estimate low-dose risk by linear extrapolation from a POD. These models also have uncertainties related both to the linear assumption and to the slope that results from linear extrapolation from a POD. However, arguments for the use of linearity have scientific support in many situations such as when the toxic substance or a metabolite acts by interacting with DNA or by augmenting a mechanism that is already acting to produce background responses (e.g., Crump et al. 1976). Regarding the slope that results from linear extrapolation from a POD, except in the case of concave dose–response curves, it is generally expected that such a slope will be a reasonable upper bound to the actual slope at low dose. Even this limited amount of scientific support appears to be missing from HVD modeling. It should also be noted that if HVD modeling is not restricted to the log-normal distribution, it is not an alternative to linear extrapolation. This is because distributions exist, including those that match the log-normal distribution closely in the observable range, that are linear at low dose and nearly linear between a reasonably low POD and the origin.

Developing reliable estimates of low-dose risk is extraordinarily difficult. Weinberg (1972) referred to the low-dose extrapolation problem as “transscience,” meaning a problem that can be stated in scientific terms but that science is unable to answer. This sentiment appears to be just as relevant today as when originally expressed. Aside from HVD modeling, the most focused attempt at improving estimates of low-dose risks has been through biologically based dose–response (BBDR) models. Such models incorporate data on biological processes at the cellular and molecular level to link external exposure to an adverse effect. At one time there was considerable optimism that BBDR models could provide more reliable estimates of low-dose risk. Today that optimism seems misplaced, and future prospects for BBDR modeling to provide improved estimates of low-dose risk are not encouraging. In a companion paper (Crump et al. 2010), we detail the impediments in developing BBDR models for this purpose and conclude that “these problems appear so intractable . . . that BBDR models are unlikely to be fruitful in reducing uncertainty in quantitative estimates of human risk from low-level-exposures.”

Given these difficulties with BBDR and HVD modeling, we expect that linear extrapolation methods, like those proposed by the NRC committee in conceptual models 1 and 3, are likely to be the least uncertain of the quantitative approaches to assigning reasonable upper bounds on human risk. This conclusion arises out of the consideration that, with the possible exception of a population threshold response, linear is the only particular dose–response shape that has any general scientific support, and linear extrapolation from an appropriately chosen POD is generally considered to be a reliable approach for estimating its upper bound slope. Basing quantitative risk estimates on the concept of a population threshold is not appealing for two reasons. First, even in cases in which individual thresholds may exist, whether a population threshold exists will be uncertain. Second, even if a population threshold were to exist, it would generally not be possible to set bounds for its value without making unverifiable assumptions.

Presently there is optimism that advances in molecular toxicology that permit many more chemicals to be tested in *in vitro* systems at greatly accelerated rates and reduced costs (Collins et al. 2008; NRC 2007) will revolutionize toxicity testing and risk assessment. The strength of these methods arises from their ability to provide information on more proximal markers of dose and on early markers of contributions from multiple pathways to diseased states (Andersen and Krewski 2009; Krewski et al. 2009). Although these *in vitro* systems may have increased sensitivity that will allow the observable range to extend to lower doses, at the same time they will require “new” extrapolations, such as from *in vitro* to *in vivo* and from early marker to adverse effect, in order to generate quantitative estimates of human risk. Given the difficulty in incorporating *in vivo* data on intermediate steps in toxic processes into BBDR models that can reliably be used to predict human risk, we are pessimistic about the prospects of using *in vitro* data effectively in such models. We believe that the potential of these data for characterizing human health risks (and thus providing support for setting exposure standards to protect human health), while reducing the need for whole animal testing, can best be realized through risk assessment and risk management paradigms that do not involve or require developing quantitative estimates of low-dose human risk.

## Figures and Tables

**Table 1:** Comparison of fits to data^a^ on variability in pharmacokinetic variables.

Model	No. data sets fit best by model^b^	Total data sets (%)
Log normal	38	19
Gamma	77	39
Log gamma	83	42
Total	198	100

a From database files 1–4 (Hattis 2006).

b Based on the AIC (Akaike 1974).

**Table 2:** Comparison of risks predicted by Expression 7 with *p*_0_ = 0.05, σ = 0.4, and *m*(*D*) = 1 + *D*^k^ with corresponding risks predicted by Expression 1.

Dose	Risks from Expression 7			Risks from Expression 1
	*k* = 0.5	*k* = 1	*k* = 2	
POD	0.5	0.5	0.5	0.5
POD/10	1.8 × 10^−1^	5.0 × 10^−2^	4.6 × 10^−3^	6.2 × 10^−3^
POD/10	5.0 × 10^−2^	4.6 × 10^−3^	4.5 × 10^−5^	2.9 × 10^−7^

## Appendix Additional Details on Mathematical Expressions

### Expression 4

We write *X = X(D)* to indicate the dependence of the apical response *X* upon dose *D*. Since *X(D)* ≥ *A* is equivalent to *D**_T_* ≤ *D*, we can write

### Expression 6

If *D* in the right-hand side of Expression 5 is replaced by POD, the left-hand side equals 0.5, which yields an equation involving POD and *A*. If this equation is solved for *A* and the result substituted for *A* in Expression 5, then Expression 6 results.

### Expression 7

If *m*(*D*) in the right-hand side of Expression 5 is replaced by *m*(0), the left-hand side equals 0, which yields an equation involving *m*(0) and *A*. If this equation is solved for *A* and the result substituted for *A* in Expression 5, then Expression 7 results.

### Expression 9

In this model the apical response *X* has a compound distribution (Johnson et al. 1999) specified in terms of a log-normal distribution (Expression 8) that depends on the parameter *T*, which has its own distribution. For a fixed half-life *T* = *t*, the blood concentration is *C* = *KDt*. Consequently, corresponding to Expression 8,

Moreover, the half-life *T* has its own distribution with log-normal probability density function

Consequently,

### Expression 10

This expression is derived in the same way as Expression 9, but using Expression 6, with *D* replaced by *C*, POD replaced by POC, and σ replaced by σ_1_, in place of Expression 8.
